# Engineering Strategies of Colloidal Drug Delivery Systems, a Rising Star in Ocular Neovascular Diseases

**DOI:** 10.3390/molecules31071169

**Published:** 2026-04-01

**Authors:** Yueping Bai, Lu Liu, Cui Li, Yiping Ma, Pai Liu, Shuo Wang

**Affiliations:** 1 Tianjin Key Laboratory of Ophthalmology and Visual Science, Tianjin Eye Hospital, Tianjin 300020, China; 2 State Key Laboratory of Medicinal Chemical Biology, College of Pharmacy and KLMDASR of Tianjin, Nankai University, Tongyan Road, Haihe Education Park, Tianjin 300350, China; 3 State Key Laboratory of Advanced Separation Membrane Materials, School of Materials Science and Engineering, Tiangong University, Tianjin 300387, China; 4 Cangzhou Institute of Tiangong University, Cangzhou 061000, China; 5 Nankai University Affiliated Tianjin Eye Hospital, Nankai University Optometry and Vision Science Institute, Tianjin 300071, China

**Keywords:** ocular neovascular diseases, colloidal drug delivery systems, stimulus-responsive nanomedicine, ocular physiological barriers

## Abstract

Ocular neovascular diseases represent a major cause of irreversible vision loss worldwide, while the complex ocular barrier system significantly limits the efficacy of conventional treatment approaches. In this context, colloidal drug delivery systems (CDDSs) have emerged as an innovative nanomedicine strategy that demonstrates remarkable advantages in enhancing ocular drug bioavailability and treatment precision through the integration of sustained release, active targeting, and stimulus-responsive functional modules. This review systematically summarizes recent research advances in CDDSs for treating ocular neovascular diseases, with a particular focus on design strategies and mechanisms for overcoming physiological barriers and achieving lesion-specific drug delivery. Furthermore, it provides in-depth analysis of key challenges in current clinical translation. With ongoing technological advancements, CDDSs are expected to offer breakthrough solutions for treating ocular neovascular diseases, ultimately leading to significant improvements in patients’ visual prognosis and quality of life.

## 1. Introduction

Ocular neovascular diseases, consisting of diabetic retinopathy (DR), age-related macular degeneration (AMD), and retinopathy of prematurity (ROP), are deemed to be the main causes of irreversible blindness worldwide [1,2,3,4]. Even though they present differences in clinical terms, they still share a common pathological hallmark, which is named pathological neovascularization. Pathological neovascularization is driven by interrelated mechanisms, including dyslipidemia, inflammation, and retinal ischemia/hypoxia [5,6,7]. These processes converge to induce persistent overexpression of pro-angiogenic factors like VEGF, leading to neovascularization and blood–retinal barrier disruption. The global burden of these diseases is substantial and escalating. DR is the primary cause of preventable blindness among working-age adults, affecting approximately 103 million people in 2020 figures and is projected to rise to 160 million by 2045 [8]. Meanwhile, neovascular AMD has emerged as the leading cause of irreversible vision loss in the elderly population in developed countries, with its global prevalence expected to increase from 211 million cases in 2023 to 288 million by 2040 [9]. Furthermore, ROP poses a critical threat to pre-term infants, with incidence rates reaching as high as 73% in extremely pre-term cohorts [10,11]. This converging epidemic of neovascular eye diseases underscores an urgent and growing unmet medical need, driving the quest to elucidate their underlying molecular drivers and develop targeted, effective therapies.

Ocular drug delivery remains a major challenge due to the eye’s highly specialized anatomy and multilayered physiological barriers [12,13]. The eye is anatomically divided into anterior and posterior segments, each protected by distinct structural and biochemical defense mechanisms that tightly regulate molecular transport. In the anterior segment, the corneal epithelium—characterized by tight junctions and lipophilic cell membranes—restricts paracellular diffusion, while rapid tear turnover and nasolacrimal drainage further limit drug residence time on the ocular surface [14]. For posterior segment delivery, the blood–retinal barrier (BRB), composed of tight junctions between retinal endothelial and retinal pigment epithelial cells, severely restricts systemic drug penetration into retinal tissues. Additionally, the viscoelastic vitreous matrix imposes diffusional resistance to intravitreal formulations [15]. Collectively, these barriers significantly reduce ocular bioavailability and complicate effective pharmacological intervention.

In this context, colloidal drug delivery systems (CDDSs) have emerged as promising candidates to overcome these translational challenges. CDDSs, including liposomes [16], polymeric nanoparticles [17], micelles [18], and dendrimers [19], offer distinctive advantages rooted in their nanoscale properties. As drug carriers, they enable sustained or controlled release of therapeutic agents, significantly prolonging therapeutic action and enhancing ocular bioavailability. From a clinical standpoint, these capabilities directly translate into reduced dosing frequency, minimized systemic side effects, and substantially improved patient compliance [12,20,21]. For instance, liposomes, with their biomimetic membrane structures, can encapsulate a wide range of drugs, while polymeric nanoparticles allow for tunable, biodegradable release profiles. Micelles enhance the solubility of hydrophobic compounds, and dendrimers can be surface-functionalized for active targeting, collectively representing a versatile toolkit for advanced ophthalmic pharmacotherapy [22,23,24,25].

This review will systematically elaborate on the pathological mechanisms underlying ocular neovascular diseases and provide a critical analysis of the limitations inherent in current clinical strategies. We will then focus on the intelligent design of CDDSs tailored to navigate the complex ocular physiological environment. A comprehensive summary of the latest research progress—spanning from rational material selection and functionalization to preclinical and clinical applications—will be presented. Through this exploration, we aim to provide a solid theoretical foundation and a forward-looking technical framework for pioneering the next generation of non-invasive, efficient, and long-acting therapeutic strategies against blinding ocular neovascular diseases.

## 2. Physiological and Treatment Barriers

### 2.1. Physiological Barriers

Effective drug therapy depends not only on the intrinsic activity of the pharmaceutical agent but also on its ability to overcome the intricate barrier systems within the eye and reach the pathological site at sufficient concentrations [26,27,28]. The physiological barriers of the eye protect its internal structures but also pose significant challenges for drug delivery, particularly to the posterior segment. This complex barrier system can be systematically categorized into physiological clearance barriers, static anatomical barriers, and other contributing barriers, all of which collectively restrict drug bioavailability [21,29]. For instance, dynamic clearance barriers positively operated external drugs and caused exceptionally brief corneal contact times, which only allowed approximately 1–5% of intraocular penetration [30,31]. Additionally, the cornea has a nonnegligible influence on drug permeability, which is attributed to its the three–six layers of tightly interconnected lipid-rich cells [32,33,34]. This sequential “lipophilic–hydrophilic” dual barrier structure creates a characteristic biphasic permeability profile of the cornea [35]. Consequently, effective transcorneal penetration requires drug molecules to possess precisely balanced oil–water partition coefficients and dissociation constants (pKa), combined with delivery vehicles that exhibit appropriate mucoadhesive and penetration-enhancing properties [36,37].

Last but not least, blood–retinal barrier rigorously regulate material exchange through dual mechanisms of physical obstruction and biochemical screening, which almost completely blocks the penetration of macromolecules and hydrophilic drugs from the bloodstream into the retinal tissue [38,39]. This structural characteristic results in only a few small-molecule drugs that meet specific physical and chemical parameters (e.g., molecular weight of less than 500 daltons and optimal lipophilicity) being able to undergo limited passive diffusion after systemic administration, while the vast majority of therapeutic macromolecular drugs (including protein drugs, monoclonal antibodies, and nucleic acid drugs) are completely blocked [40,41]. Studies have shown that the bioavailability of drugs in retinal tissue after systemic administration is usually less than 2% [42]. To achieve therapeutic concentrations, a significant increase in dose is often required, which leads to systemic exposure and associated non-targeted toxicity risks.

In consideration of physiological barriers, even though the injection of anti-VEFG drug is common and useful for ocular neovascular diseases, the frequency of injections should be kept as every 4 to 8 weeks [43]. These frequent invasive injections not only heighten the psychological and financial burden on patients but also raise the risk of a range of complications, such as endophthalmitis, retinal detachment, vitreous hemorrhage, and iatrogenic cataract [44,45].

### 2.2. Sensitively Pathophysiological Microenvironment

As one of the tissues with the highest oxygen consumption, the neurovascular unit of the retina is extremely sensitive to fluctuations in oxygen supply [46]. In pathological conditions such as DR, severe hypoxia caused by microvascular occlusion is the initial factor for neovascularization. This local hypoxic microenvironment leads to the rapid stabilization and accumulation of hypoxia-inducible factor-1α (HIF-1α) by inhibiting the ubiquitin–proteasome pathway, which in turn activates the transcriptional expression of downstream VEGF and drives pathological angiogenesis [47,48]. Under stress conditions such as high glucose toxicity or light damage, the generation rate of ROS in the retina far exceeds the clearance threshold of the endogenous antioxidant system [49]. Excessive superoxide anion and hydrogen peroxide not only directly damage the DNA and proteins of photoreceptor cells and induce apoptosis but also act as key signaling molecules to activate the NF-κB and NLRP3 inflammasome pathways, building a vicious cycle of “oxidative stress–inflammation–angiogenesis” [50]. In this process, NF-κB activated by ROS rapidly translocates into the nucleus, directly up-regulates the transcriptional expression of VEGF, intercellular adhesion molecule-1 (ICAM-1) and angiopoietin-2 (Ang-2), and initiates angiogenesis at the genetic level [51,52]. At the same time, interleukin-1β (IL-1β) and TNF-α, which are released by NLRP3 inflammasomes, act synergistically to induce pathological adhesion (Leukostasis) between leukocytes and retinal vascular endothelial cells [53,54]. This extensive leukocyte arrest not only blocks the capillaries and further aggravates local ischemia and hypoxia but also degrades the tight junction proteins between endothelial cells (such as Occludin and ZO-1) by secreting proteases, leading to the collapse of the blood–retinal barrier (BRB) and the sharp increase in vascular permeability, and finally promotes the explosive growth of pathological neovascularization [55,56].

Undeniably, MMP-2 (gelatinase A) and MMP-9 (gelatinase B) expression levels and enzyme activities are significantly up-regulated in the pathological lesions of choroidal neovascularization (CNV) and DR [57]. They not only directly destroy the vascular basement membrane scaffold by specifically hydrolyzing collagen type IV and laminin but also release the bound pro-angiogenic factors, such as VEGF, from the ECM reservoir, thus clearing the physical obstacles and providing biochemical induction for the directional invasion and migration of vascular endothelial cells [58,59].

### 2.3. Irreversible Damage After Treatment

Besides the injection of anti-VEFG, other treatments, such as laser photocoagulation, photodynamic therapy and pars plana vitrectomy, all have different advantages and disadvantages [60,61,62]. As the earliest standard, the core mechanism of laser photocoagulation is to irradiate the focal area of the retina with a high-energy laser beam to cause thermal coagulation of local tissues, thereby sealing abnormally leaking blood vessels, reducing tissue exudation, and inhibiting subsequent neovascularization [63]. However, laser action is irreversible, and its thermal damage often spreads to the surrounding normal retinal tissue, leading to permanent visual field defects, abnormal color vision or dark adaptation, and other dysfunctions [64]. Meanwhile, laser therapy can cause severe visual loss in patients with foveal macular area or lesions located in functionally sensitive areas, so its clinical application is strictly limited [65,66].

In comparison with laser photocoagulation, photodynamic therapy can better preserve the retinal neuroepithelial structure, but with the accumulation of long-term medical evidence, its biophysical limitations and potential toxicity have become increasingly prominent [61]. First, PDT carries a significant risk of inducing or accelerating retinal pigment epithelial (RPE) Atrophy and Macular Atrophy. Clinical studies have shown that long-term ischemia of choroidal capillaries caused by photochemical reactions can lead to secondary degeneration of RPE [67,68]. Due to the non-uniform distribution of photosensitizer in tissues and the diffusion effect of reactive oxygen species (ROS), the treatment process may be accompanied by non-specific oxidative damage to the surrounding normal blood vessels and nerve tissues [69,70]. In view of these limitations, current international clinical guidelines have downgraded it from first-line treatment of most ocular neovascular diseases, and it is more often used as a combination/salvage treatment for specific subtypes (such as polypoidal choroidal vasculopathy) or patients with poor response to anti-VEGF therapy [67,68].

Under serious conditions, pars plana vitrectomy (PPV) is deemed a salvage treatment, especially in tractional retinal detachment (TRD) and non-clearance vitreous hemorrhage due to fibrovascular membrane proliferation [71]. PPV can rapidly restore the transparency of the refractive stroma, relieve the mechanical traction of the retinal surface, and promote the anatomical reattachment of the retina by microsurgical removal of the cloudy vitreous body and the peeling-off of the pathological proliferative membrane [62,71]. Since the driving mechanism of VEGF cannot be fundamentally blocked, some patients still face the risk of recurrence or the rebleeding of neovascularization after vitrectomy (i.e., vitreous hemorrhage after vitrectomy, POVFH), and treatment often needs to be combined with panretinal photocoagulation or anti-VEGF drugs for long-term maintenance treatment [72].

## 3. Coping Strategy of Colloidal Drug Delivery Systems

To address the limitations of conventional therapies, the design of CDDSs has evolved from simple drug carriers to intelligent bio-interactive platforms. This shift is exemplified by stimulus-responsive systems, which achieve precise spatiotemporal control over drug release by recognizing characteristic biochemical signals within pathological microenvironments [73,74,75], such as elevated ROS, specific enzyme overexpression, and abnormal pH levels. In response to these endogenous stimuli, CDDSs undergo controlled structural or physicochemical transformations, thereby converting pathological features into biological triggers for drug release. This allows for autonomously activated drug delivery precisely at lesion sites while remaining inactive in healthy tissues, significantly enhancing therapeutic efficacy and minimizing off-target effects [76,77,78]. The following section systematically elaborates on various CDDS platforms based on their targeted physiological challenges, highlighting this paradigm shift from passive drug loading to active, disease-responsive regulation.

### 3.1. Controlled-Release CDDSs

Based on the description of physiological barriers, it is believed that efficient dynamic clearance mechanisms of the eye-including tear turnover, nasolacrimal drainage, and aqueous humor circulation, severely limit drug residence time, resulting in exceptionally low ocular bioavailability [15,79,80]. Sustained-release colloidal drug delivery systems are specifically designed to counteract these rapid elimination processes by prolonging the duration of therapeutic drug concentrations at the target site. For instance, lipid-based nanocarriers such as lipid nanocapsules (LNCs) and nanostructured lipid carriers (NLCs) have demonstrated remarkable potential in this regard. LNCs, characterized by a hydrophobic core and a non-ionic surfactant shell, enable a high loading of hydrophobic drugs and enhance their stability and bioavailability [81,82]. Bohley et al. reported that cyclosporin A-loaded LNCs facilitated targeted delivery to retinal pigment epithelial cells via lipoprotein receptor interactions, effectively suppressing pathological neovascularization and inflammation in a ROP model following a single intravenous injection [83]. Similarly, Tan et al. developed a composite system based on NLCs loaded with dexamethasone (DEX), which was further functionalized with chondroitin sulfate conjugated to (3-aminomethylphenyl)boronic acid (APBA-ChS). This design effectively prolonged corneal retention and enhanced drug delivery performance [84]. Beyond lipid systems, polymer-based nanoparticles also offer extended release profiles [85]. Pandit’s group developed chitosan-coated PLGA nanoparticles (CS-PLGA NPs) for the delivery of the vascular endothelial growth factor inhibitor bevacizumab. Leveraging the mucoadhesive and penetration-enhancing properties of chitosan, the system effectively prolonged ocular retention and improved the efficiency of drug delivery to posterior ocular tissues [86]. More impressively, Yandrapu reported that bevacizumab-loaded PLGA nanoparticles-in-porous-microparticles (NPinPMPs) maintained stable drug release for up to four months in vivo [87]. Collectively, these advanced systems exemplify the successful application of sustained-release CDDSs in overcoming ocular dynamic clearance, thereby extending therapeutic efficacy and reducing the need for frequent administration.

**Figure 1:**
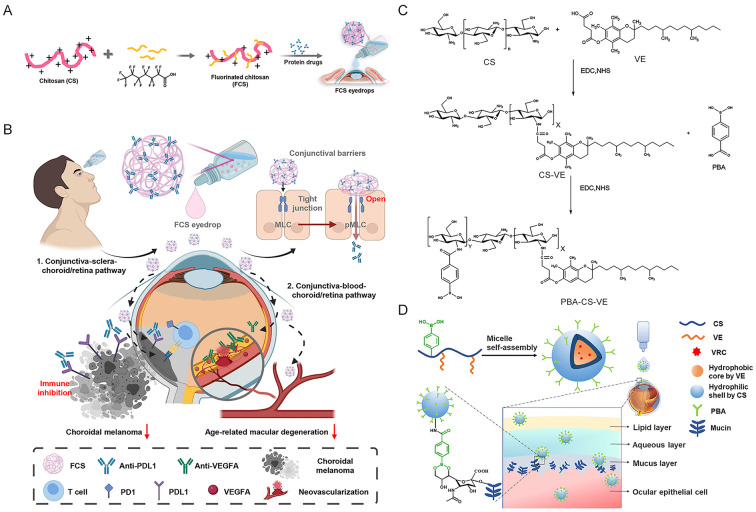
(**A**) Illustration of the synthesis of FCS/IgG nanocomplexes. (**B**) Schematic illustration of FCS as the ocular barrier penetration carrier for effective macromolecular delivery for fundus disease treatment. Copyright 2023, The American Association for the Advancement of Science. (**C**) The synthesis route of phenylboronic acid-conjugated chitosan oligosaccharide–vitamin (PBA-CS-VE) copolymer. (**D**) Schematic illustration of PBA-CS-VE-VRC nanomicelles adhering to the mucosa and prolonging ocular surface retention. Copyright 2022, Elsevier Ltd.

Besides long-time release, CDDSs have been rationally engineered with enhanced penetration capabilities and active targeting functionalities. Representative systems leveraging endogenous biomolecules and functional polymers demonstrate particular promise [88,89,90]. Cationic polysaccharides exploit their positive charge to interact with negatively charged ocular surfaces. Shen et al. developed fluorocarbon-modified chitosan (FCS) that loosens tight junctions between corneal and conjunctival tissues (Figure 1A), enabling non-invasive delivery of anti-VEGF proteins to the posterior segment. Topical administration of FCS-based nanocomplexes demonstrated superior efficacy over intravitreal injections in choroidal neovascularization models, highlighting its transformative potential for posterior segment therapy (Figure 1B). Albumin-based nanoparticles, capitalizing on the protein’s innate ligand-binding capacity and biocompatibility, can be functionalized for active transport [91]. For instance, Koo et al. demonstrated that intravitreally injected HSA-NPs have been shown to interact with Müller cells, disrupt the inner limiting membrane, and successfully traverse the entire retina to reach retinal pigment epithelial cells [92]. Similarly, hyaluronic acid (HA), with its inherent affinity for CD44 receptors, serves as an excellent targeting moiety. Surface-engineered HA nanoparticles significantly enhance corneal permeation, as demonstrated by imatinib-loaded systems that effectively crossed the corneal barrier and inhibited pathological endothelial sprouting [93]. Further extending this approach, Li et al. developed an HA-grafted copolymer (MPEG-b-PAE-g-HA) that encapsulates genistein, forming nanoparticles with enhanced corneal penetration, sustained-release profile, and significant anti-angiogenic activity.

Beyond endogenous ligands, synthetic functionalization offers another powerful strategy [94]. Sun et al. [95] developed mucin-binding PBA-CS-VE nanospheres (Figure 1C) for loading the antifungal drug voriconazole (VRC) and preparing adhesive nanospheres for the treatment of fungal keratitis. This material exhibits excellent mucosal adhesion properties. Permeability experiments using HCE-T cell monolayers and three-dimensional cell spheroids confirmed that it effectively enhances corneal drug penetration by modulating intracellular calcium ion concentration, cell membrane potential, membrane fluidity, and intercellular junctions. Pharmacokinetic analysis of tear fluid showed that these nanospheres significantly prolong ocular drug retention and improve corneal penetration efficiency. In a rabbit model of fungal keratitis, voriconazole-loaded PBA-CS-VE (PBA-CS-VE-VRC) nanospheres demonstrated superior therapeutic efficacy compared with the free drug (Figure 1D). Collectively, these engineering approaches—whether utilizing natural targeting pathways or synthetic modifications—enable CDDSs to navigate the intricate static barriers of the eye, facilitating precise drug delivery to otherwise inaccessible pathological sites.

### 3.2. ROS-Responsive CDDSs

ROS-responsive nanocarriers represent a class of intelligent drug delivery systems designed to exploit the elevated oxidative stress characteristic of pathological ocular microenvironments [96,97,98,99]. These systems incorporate chemical motifs—such as thioketal [100], boronic ester [101], or selenium-containing groups [102]—that undergo selective cleavage or structural transformation upon ROS exposure, enabling spatiotemporally controlled drug release at diseased sites.

**Figure 2:**
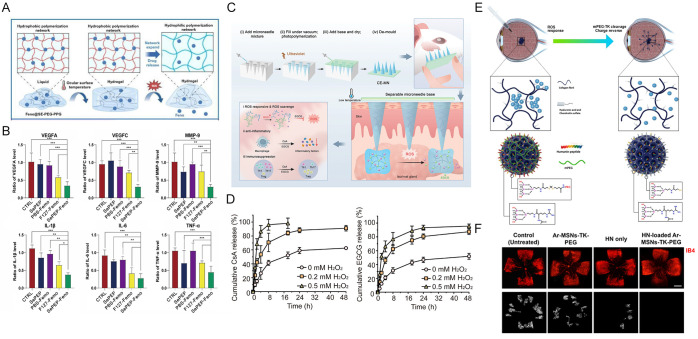
(**A**) Schematic illustration of Se-PEG-PPG (SePEP) for the treatment of CNV and various ROS-related ocular diseases. (**B**) Detection of gene expression levels of angiogenic factors in corneal tissue of each group. VEGFA, VEGFC, MMP-9, IL-1β, IL-6, and TNF-α normalized to GAPDH level. Significance levels are indicated by ns (not significant, *p* ≥ 0.05), * *p* < 0.05, ** *p* < 0.01, and *** *p* < 0.001. Copyright 2025, Elsevier B.V. (**C**) Schematic illustration of the fabrication process and therapeutic mechanism of the ROS-responsive separable MN patches for SSDE therapy. (**D**) Cumulative in vitro release of CsA and EGCG from the CE-MN 0.01 system at different concentrations of H_2_O_2_. Copyright 2024, Wiley-VCH. (**E**) Modified MSNs with a partial positive charge are trapped within the vitreous gel. (**F**) Immunostaining of retinal whole-mounts at P17 shows neovascularization (indicated by white dots) in untreated, HN-only, Ar-MSNs-TK-PEG, and HN-loaded Ar-MSNs-TK-PEG retinas, using Isolectin B4 (red) as a marker. Scale bars: 500 μm. Copyright 2025, Elsevier B.V.

In recent research, Xiang et al. developed a dexamethasone-loaded ROS-responsive nanogel (DEX@INHANGs) to overcome the limitations of conventional dexamethasone eye drops. This system is constructed as a supramolecular network based on cyclodextrin-adamantane host–guest interactions, with the incorporation of thioketal as an ROS-responsive unit enabling controlled drug release in pathological oxidative stress environments. Following modification with integrin β1-fusion protein, the nanogel demonstrates significantly prolonged retention time on the corneal surface and effectively enhanced bioavailability [96]. Similarly, Fan et al. developed a selenium-containing thermosensitive hydrogel (Se-PEG-PPG, SePEP), which exhibits dual functionality: it forms a gel at ocular surface temperature to enhance corneal adhesion and residence time, while its selenium-based structure responds to and scavenges pathological ROS (Figure 2A). Loaded with the insoluble drug fenofibrate, this system achieves sustained drug release and synergistically activates the NRF2 signaling pathway with its payload, effectively alleviating oxidative stress and inflammation. This strategy provides an integrated antioxidant and anti-angiogenic therapy for CNV (Figure 2B) [103].

Expanding the application scope of ROS-responsive systems, Mu et al. developed a detachable ROS-responsive microneedle patch (CE-MN) for periorbital administration. This sophisticated platform penetrates periocular skin to deliver therapeutic agents directly to the lacrimal gland region (Figure 2C). The system incorporates multiple smart elements: epigallocatechin gallate serves dual roles as both a therapeutic agent and a crosslinker, while a thermosensitive polymer enables controlled separation of the needle base post-insertion. The CE-MN patch responds to ROS levels to regulate drug release, maintaining therapeutic concentrations in the lacrimal gland for over 48 h—significantly longer than conventional eye drops (Figure 2D). In dry eye models, this system demonstrated potent anti-inflammatory and immunomodulatory effects through ROS scavenging and suppression of pathogenic immune cells [104].

In another innovative approach extending this design philosophy, Elbedwehy’s group designed a smart delivery system based on mesoporous silica nanoparticles (MSNs) for the responsive delivery of humanin (HN) to retinal pigment epithelial cells (ARPE-19). The core pore size of the system was designed to be 2.8 nm, achieving an HN loading capacity of up to 64.4% (*w*/*w*). The outer surface was modified with 20% acetyl-L-arginine (Ar) to introduce a partially positive charge, while 80% of the surface was covalently linked with thioketal (TK)-conjugated methoxy polyethylene glycol (mPEG) as a ROS-responsive “gating” molecule. The constructed Ar-MSNs-TK-PEG (initial average zeta potential: +2 mV) can utilize its weak positive charge to be retained in the negatively charged vitreous matrix (Figure 2E). Under oxidative stress conditions, the cleavage of TK bonds leads to the shedding of mPEG, shifting the nanoparticle surface charge to −25 mV, thereby promoting the diffusion of HN-loaded nanoparticles toward the retina. Cell experiments demonstrated that ARPE-19 cells efficiently internalize HN-loaded Ar-MSNs-TK and release the active peptide, significantly alleviating oxidative stress-induced apoptosis. Furthermore, in an oxygen-induced retinopathy (OIR) mouse model, this system markedly inhibited pathological retinal neovascularization (Figure 2F) [105].

### 3.3. Enzyme-Responsive CDDSs

Enzyme-responsive colloidal drug delivery systems represent an advanced strategy that exploits the characteristic enzyme overexpression in pathological ocular microenvironments to achieve targeted drug release [106,107]. These intelligent carriers incorporate specific peptide substrates that remain stable during circulation but undergo rapid cleavage upon encountering the pathological enzyme milieu, resulting in site-specific drug release at active neovascular lesions [108].

**Figure 3:**
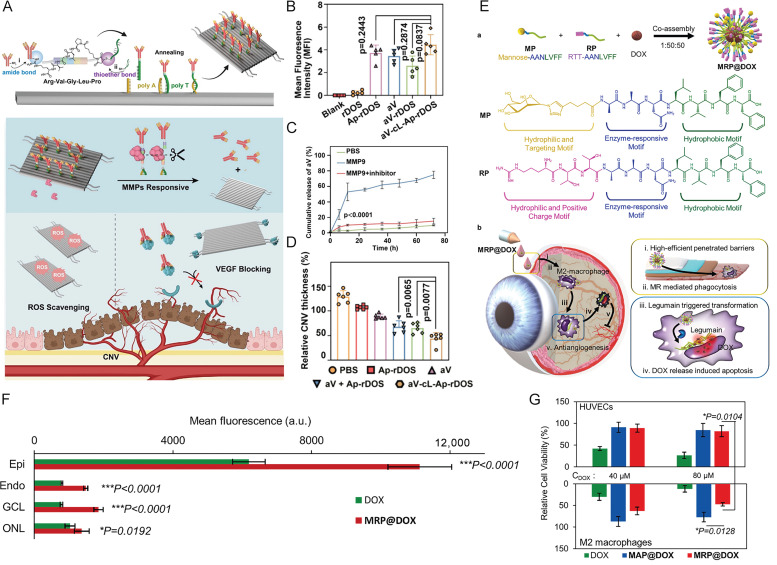
(**A**) Schematic illustration of the V-cL-Ap-rDOS assembly and its mechanism of action. (**B**) Levels of aV released from aV-cL-Ap-rDOS after different treatments, as indicated. aV-cL-Ap-rDOS was treated with MMP9 or MMP9 + inhibitor. The aV levels were quantified by ELISA. Significance levels are indicated by ns (not significant, *p* ≥ 0.05), * *p* < 0.05, and *** *p* < 0.001. (**C**) The corresponding mean fluorescence intensity (MFI) of CECs incubated with blank, rDOS, Ap-rDOS, aV, aV-cL-rDOS, or aV-cL-Ap-rDOS (10 nM, 100 μL) for 2 h at 37 °C. (**D**) Corresponding statistical analysis of CNV thickness in the H&E staining of retinal slices from CNV-bearing mice after intravitreal injection of PBS, Ap-rDOS, aV, aV + Ap-rDOS, aV-fL-Ap-rDOS, or aV-cL-Ap-rDOS. Significance levels are indicated by ns (not significant, *p* ≥ 0.05), * *p* < 0.05, and *** *p* < 0.001. Copyright 2024, American Chemical Society. (**E**) Scheme 1. (a) Schematic illustration of co-assembled components of MRP@DOX, which constructed as glycopeptide nanotransformers (GPNTs). The chemical structures of amphiphilic peptides of MP and RP with modular division. Hydrophobic motif (green): LVFF; Enzyme-response motif (blue): AAN; Hydrophilic and targeting motif (yellow): Mannose; Hydrophilic and positive charge motif (purple): RTT. (b) The five working procedures: (i) cornea and sclera barrier penetration; (ii) mannose receptor (MR) targeting mediated internalization into M2 macrophages; (iii) legumain induced transformation instructed lysosome escape; (iv) nanofibrous DOX retention enhanced M2 macrophages apoptosis; (v) M2 macrophages elimination strengthened antiangiogenesis. (**F**) The cytotoxicity of HUVECs and M2 macrophages with the same equivalent of DOX. After being treated with DOX, MAP@DOX, and MRP@DOX for 1 h, fresh drug-free culture medium was replaced every 3 h, and cell viability was detected at 24 h. (**G**) The quantitative calculation of the fluorescence per area in the layers of Epi, Endo, GCL and ONL. Copyright 2022, Elsevier Ltd.

Wu et al. recently reported a groundbreaking enzyme-responsive DNA origami–antibody conjugate platform developed for combined therapy of choroidal neovascularization. This sophisticated system utilizes a rectangular DNA origami structure conjugated with anti-VEGF antibodies through matrix metalloproteinase (MMP)-cleavable peptide linkers while simultaneously incorporating VEGF aptamers on its surface (Figure 3A). In experimental models, the construct demonstrates dual-targeting capability through both antibody and aptamer interactions, leading to specific accumulation at neovascular lesions (Figure 3B). Upon exposure to the MMP-rich microenvironment, the peptide linkers undergo enzymatic cleavage, resulting in controlled antibody release (Figure 3C). The liberated antibodies and surface aptamers work synergistically to inhibit VEGF activity, while the residual DNA origami framework functions as an effective reactive oxygen species scavenger, collectively achieving synergistic suppression of neovascularization through combined anti-angiogenic and antioxidant mechanisms (Figure 3D) [109].

An alternative strategy developed by Ke Li and colleagues involves the enzyme-responsive co-assembled glycopeptide nanotherapeutic system (GPNT). This nanoplatform is constructed through the co-assembly of glycopeptides and cationic peptides, followed by doxorubicin loading (Figure 3E). The system leverages its positive surface charge to prolong ocular surface retention through interaction with the negatively charged mucin layer, while facilitating penetration across corneal and scleral barriers (Figure 3F). Once it reaches the posterior segment, the nanoconstruct specifically targets M2-type macrophages and undergoes legumain enzyme-triggered transformation from nanoparticles to nanofibers. This structural transition enables sustained doxorubicin release, effectively inducing apoptosis in M2 macrophages and consequently inhibiting ocular neovascularization (Figure 3G) [110].

Among inorganic platforms, mesoporous silica nanoparticles (MSNs) have emerged as one of the most versatile carriers for enzyme-responsive ocular drug delivery owing to their highly ordered mesoporous structure, tunable pore sizes (typically 2–10 nm), and amenability to surface functionalization. Unlike polymeric carriers, the hard skeleton and regular pore structure of MSNs enable them to load macromolecular drugs, such as macromolecular inhibitors, and maintain structural stability and high loading capacity [111,112,113]. A systematic review by Sun et al. emphasized that the incorporation of enzyme-responsive molecular gatekeepers at MSN pore entrances enables precise spatiotemporal control of drug release. Such designs effectively suppress background drug leakage in the vitreous and non-pathological retinal tissues while allowing selective activation in disease-specific enzymatic microenvironments [114]. Building on this design principle, Vaghasiya and co-workers reported an MMP-2-responsive gate-controlled MSN system [115]. In this system, MMP-cleavable PLGLAG peptide linkers were covalently anchored at the MSN pore openings and further capped with polymeric moieties to physically block drug diffusion under low enzymatic activity. In vitro release studies demonstrated that elevated MMP-2 concentrations induced rapid peptide cleavage, pore opening, and accelerated drug release, whereas negligible leakage occurred under enzyme-deficient conditions, confirming a highly enzyme-dependent release profile. Although initially evaluated in tumor-related models, this gate-controlled strategy closely mirrors the pathological characteristics of ocular neovascular diseases, where persistent overexpression of MMP-2/MMP-9 and ECM degradation are well documented, underscoring its translational relevance.

More recently, Ultimo et al. [116] specifically addressed the development of enzyme-responsive MSN platforms tailored for ophthalmic drug delivery and therapy. The authors highlighted that MSNs can efficiently encapsulate a broad range of therapeutic cargos, including small-molecule anti-angiogenic agents, anti-VEGF proteins, and nucleic acid therapeutics. Surface PEGylation was shown to reduce non-specific adsorption and premature clearance in the vitreous humor, while additional functionalization with endothelial-targeting peptides (e.g., RGD motifs) or ECM-affinitive ligands further enhanced cellular uptake following enzyme-triggered activation.

Based on the unique physiological and pathological enzymatic milieu of the eye, such as the abundance of lysozyme in tears, enzyme-responsive CDDSs—particularly those based on MSN architectures—exemplify a rational and highly adaptable design paradigm for ECM-targeted therapy in ocular neovascular diseases [117,118]. Their strong congruence with disease-associated enzymatic signatures, combined with precise release control and broad cargo versatility [119], positions these systems among the most promising candidates for translational precision nanomedicine in ophthalmology.

### 3.4. pH-Responsive CDDSs

Maintaining an appropriate pH environment is crucial to normal ocular physiological functions. Healthy tears typically maintain a pH range of 6.5 to 7.6, while pathological conditions often lead to pH fluctuations [120]. This characteristic provides an important foundation for developing intelligent drug delivery systems. Research indicates that tear buffer capacity is closely associated with dry eye monitoring, while pH variations can significantly influence intraocular drug permeability [121].

**Figure 4:**
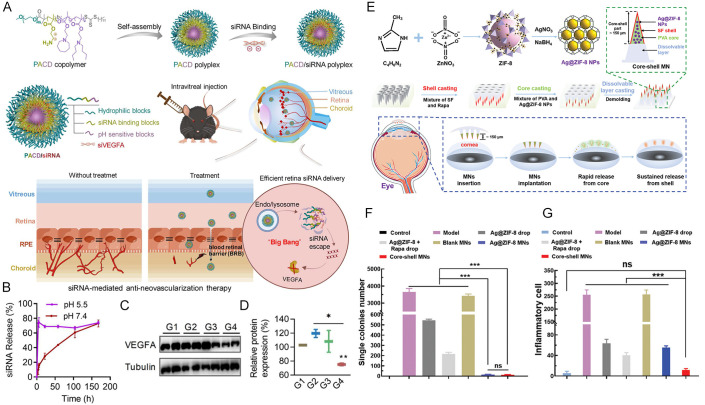
(**A**) Schematic illustration of PACD and PACD/siRNA polyplex preparation and its mechanism of action. (**B**) The release profile of siRNA at pH 7.4 and 5.5. (**C**,**D**) Western blotting images and quantification of VEGFA protein expression in each group. The blotting bands were quantified by Image J software. G1: PBS; G2: Ranibizumab; G3: PACD/siNC; G4:PACD/siVEGFA. Significance levels are indicated by ns (not significant, *p* ≥ 0.05), * *p* < 0.05, ** *p* < 0.01, and *** *p* < 0.001. Copyright 2024 Elsevier B.V, on behalf of Chinese Pharmaceutical Association and Institute of Maternal Medicine, Chinese Academy of Medical Sciences. (**E**) The schematic illustration of preparation and application of core–shell MN patches. (**F**) Quantification of bacterial colonies in each group. Significance levels are indicated by ns (not significant, *p* ≥ 0.05), * *p* < 0.05, ** *p* < 0.01, and *** *p* < 0.001. (**G**) The inflammatory cell count in the Masson staining of each group. Copyright 2024, Wiley-VCH.

Based on this principle, researchers have developed various pH-responsive delivery systems [122,123,124]. For instance, Kouchak et al. constructed a pH-sensitive hydroxypropyl methylcellulose system that effectively delivers dorzolamide hydrochloride, enhancing mucoadhesion while maintaining corneal permeability, thereby offering a novel approach for glaucoma treatment [125]. In the field of nanocarriers, Guo et al. designed a pH-responsive triblock copolymer PACD. This polymer comprises a hydrophilic PEG segment, an siRNA binding domain, and a pH-sensitive segment that undergoes protonation in acidic environments (Figure 4A). It can self-assemble into nanomicelles in aqueous solution and forms complexes with siRNA through electrostatic interactions. The delivery system remains stable under physiological pH conditions but rapidly dissociates and releases its siRNA payload in acidic environments below pH 6.2 (Figure 4B). Experimental evidence confirms that this system demonstrates excellent retinal delivery efficiency, gene silencing activity, and anti-angiogenic efficacy both in vitro and in vivo (Figure 4C,D) [126]. Expanding the application scope of this strategy, Jiang et al. reported an implantable core–shell-structured microneedle patch with programmable drug release functionality for the treatment of bacterial keratitis. This system incorporates pH-responsive antibacterial nanoparticles (Ag@ZIF-8) into the rapidly dissolvable microneedle core, while the anti-angiogenic drug rapamycin is encapsulated in the biodegradable microneedle shell layer. Upon corneal implantation, the core layer rapidly releases Ag@ZIF-8 nanoparticles, which locally release antibacterial metal ions and induce bacterial oxidative stress at the infection site; the shell layer gradually degrades, enabling sustained release of rapamycin (Figure 4E). In a rat model of bacterial keratitis, a single administration of this core–shell microneedle patch demonstrated favorable antibacterial efficacy, with superior anti-angiogenic and anti-inflammatory effects compared with conventional daily eye drop treatment (Figure 4F,G) [127].

### 3.5. Multifunctionally Integrated CDDSs

Current research paradigms in colloidal drug delivery systems are shifting from traditional single-function optimization toward innovative strategies based on multifunctional integration [128,129,130]. The core of this transformation lies in combining sustained release, precise targeting, and intelligent responsiveness within a unified nanoplatform, achieving therapeutic effect maximization through systematic collaboration among functional components. This integrated design generates remarkable synergistic enhancement effects: the targeting module significantly increases local concentration at the lesion site, establishing a foundation for prolonged therapy; stimulus-responsive mechanisms ensure that drug release occurs within the most therapeutically valuable spatiotemporal window, preventing ineffective diffusion [131,132]; and sustained, controlled drug release maintains a stable therapeutic concentration range [133]. The synergistic interaction among functional modules not only enhances treatment efficacy but also reduces off-target risks through precise drug delivery, demonstrating the considerable potential of integrated CDDSs in treating complex ocular neovascular diseases.

**Figure 5:**
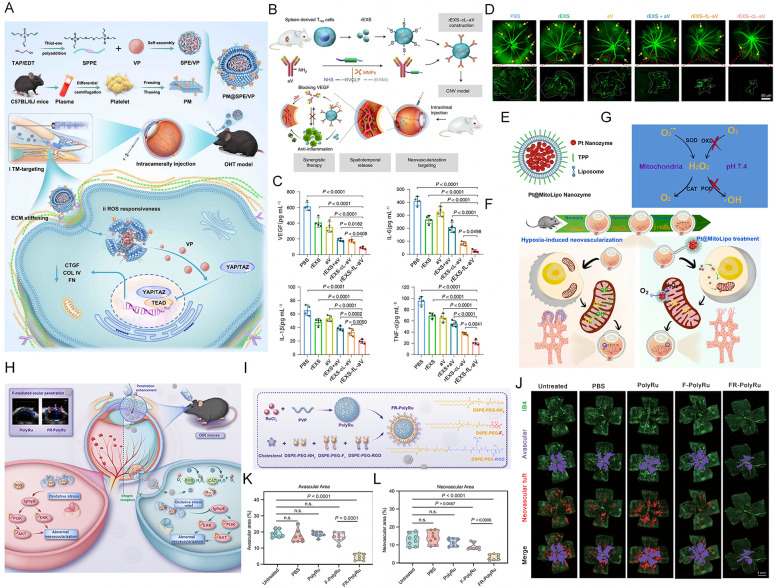
(**A**) Schematic of the synthesis procedure and glaucoma therapy of PM@SPE/VP. Copyright 2025, Wiley-VCH. (**B**) Schematic of the preparation of rEXS–cL–aV and experimental design for subsequent synergistic therapy studies in the CNV mouse model. The cleavable linker enables exosomes to release aV in response to MMP activity at CNV lesions, which can spatiotemporally regulate aV and anti-inflammation activities to suppress CNV. NH2, amine; 6-Mal, 6-maleimide. (**C**) CBA-based measurement of VEGF, IL-6, IL-1β and TNF-α levels in aqueous humor samples from CNV mice following the indicated treatments. Each sample was pooled from aqueous humor of 6 eyes from 6 mice, and 4 biological repeats were quantified in each group. (**D**) The assessment of CNV lesions by FFA. Top: Retinal arterioles and venules were visualized with fluorescein. Sites of CNV lesions are highlighted with yellow arrows. Bottom: Enlarged view of outlined regions in the top images, showing the neovascular sites. Copyright 2021 Springer Nature. (**E**) Schematic illustration of Pt@MitoLipo nanozymes. (**F**) Schematic illustration of hypoxia-induced retinal neovascularization disease and Pt@MitoLipo nanozyme-based targeted mitochondrial ROS scavenging and hypoxia relief for retinal neovascular disease therapy. (**G**) Schematic illustration of the multi-enzyme-like activities of Pt@MitoLipo nanozymes when located in mitochondria. Copyright 2022, Elsevier B.V. (**H**) Schematic illustration of the synthesis process of FR-PolyRu nanozyme. (**I**) Schematic illustration of the therapeutic effects of FR-PolyRu nanozyme. (**J**) Representative images of different groups of processed whole-mount retinas are displayed. Purple: avascular area; red: neovascular area. Scale bar: 1 mm. (**K**,**L**) Quantification of the percentage of avascular and neovascular area. Copyright 2025, The American Association for the Advancement of Science.

Recent studies have compellingly demonstrated the robust potential of integrated CDDS platforms. Sun et al. developed a platelet membrane–biomimetic ROS-responsive nanoplatform that integrates hyperbranched polyphosphoester nanoparticles with a thioketal-based ROS sensitive matrix (Figure 5A). This innovative design leverages the specific capacity of platelet membranes to bind to exposed collagen in ocular tissues, enabling significant accumulation at pathological sites following intraocular administration. By utilizing the pathological high-ROS microenvironment to trigger controlled drug release, this system achieved remarkable therapeutic outcomes with sustained efficacy, providing an innovative strategy to overcome limitations of conventional treatments for ocular disorders [134]. Tian et al. have proposed a novel strategy that surpasses existing standalone anti-VEGF antibody therapy by developing an innovative rEXS-cL-aV delivery system (Figure 5B). This system utilizes naturally anti-inflammatory rEXS as a carrier, conjugated with VEGF antibody through MMP-sensitive peptide linkers. The intelligent design leverages the inherent inflammatory tropism of rEXS to guide the antibody to neovascular lesions, where elevated MMP levels trigger controlled antibody release. By combining anti-inflammatory and anti-angiogenic effects, the system achieves synergistic therapeutic enhancement (Figure 5C,D). Experimental validation in animal models of choroidal neovascularization demonstrates that a single administration significantly suppresses disease progression, with efficacy substantially surpassing conventional anti-VEGF monotherapy [135].

In recent years, nanozymes, as artificial enzyme-mimicking materials possessing both superoxide dismutase (SOD-like) and catalase-like activities, have demonstrated remarkable capabilities in regulating oxidative stress in the field of ophthalmology [136,137,138]. Xue et al. constructed an ultrasmall (6–8 nm) platinum nanozyme-loaded mitochondrial targeted liposome (Pt@MitoLipo) (Figure 5E) for the treatment of retinal neovascular diseases by synergistically alleviating hypoxia and eliminating excess ROS (Figure 5F). The platinum nanozyme exhibits cascade catalytic activities of both superoxide dismutase and catalase, enabling the conversion of cytotoxic superoxide anions (O_2_•^−^) and hydrogen peroxide (H_2_O_2_) into non-toxic H_2_O and O_2_. By encapsulating the platinum nanozyme with triphenylphosphonium-conjugated liposomes, the system not only enhances biocompatibility but also confers capabilities for cell membrane penetration, lysosomal escape, and mitochondrial targeting, thereby achieving precise clearance of mitochondrial O_2_•^−^ and local hypoxia mitigation (Figure 5G). In an oxygen-induced retinopathy mouse model, the Pt@MitoLipo nanozyme significantly inhibited hypoxia-induced pathological neovascularization and promoted retinal vascular normalization, with no apparent toxicity observed [139]. In addition, the group also developed a more clinically feasible non-invasive treatment approach—a nanozyme-based topical eye drop delivery system. This nanozyme eye drop system can penetrate non-invasively through the ocular surface to reach the posterior segment of the eye, where it effectively scavenges ROS to inhibit neovascularization (Figure 5H). The formulation consists of liposomes formed by fluorinated and arginine–glycine–aspartate–aspartate-modified phospholipids, with surface modifications significantly enhancing its ability to penetrate ocular barriers. The liposomes encapsulate nanozymes with cascade catalytic activities of superoxide dismutase and catalase, enabling efficient ROS clearance (Figure 5I). In vitro and in vivo studies demonstrated that the eye drops effectively penetrate deep retinal tissues, alleviate local oxidative stress, restore mitochondrial function (Figure 5J–L), and inhibit pathological neovascularization by suppressing abnormal insulin-like growth factor-binding protein 6 signaling [140].

## 4. Discussion

CDDSs have emerged as cutting-edge platforms for treating ocular neovascular diseases. Through sophisticated material design strategies, they effectively overcome delivery challenges posed by the eye’s complex anatomy and physiological barriers. Traditional administration methods suffer from low bioavailability and insufficient target tissue exposure due to multiple obstacles, including the tight junctions of the corneal epithelium, rapid clearance by tear fluid, and the blood–retinal barrier. The introduction of CDDSs offers new possibilities for achieving precise, controlled drug delivery within the ocular environment. Current CDDS designs for ocular neovascular diseases no longer focus solely on basic biocompatibility and degradability requirements but are evolving toward multifunctional integration. Key dimensions for evaluating their clinical potential now include tunable physicochemical properties, drug loading efficiency, stimulus-responsive release capabilities, targeting specificity, and patient compliance. Thus, constructing next-generation high-efficiency CDDSs requires a multi-dimensional integrated design philosophy to systematically optimize these parameters and maximize therapeutic efficacy.

From a delivery efficacy perspective, CDDSs demonstrate multiple advantages in treating ocular neovascular diseases: First, sustained-release designs significantly prolong drug retention in the ocular environment, overcoming the rapid clearance limitations of conventional formulations. Second, surface-modified CDDSs enhance penetration into ocular tissues, enabling effective delivery to the posterior segment. Third, smart response systems engineered for pathological microenvironments (e.g., ROS, enzymes and pH) facilitate programmed, precise drug release at the lesion site. Additionally, conjugating targeting ligands (e.g., transferrin, HA, and RGD) confers active targeting properties to improve the therapeutic index. By integrating sustained release, targeting, and responsive mechanisms within a single platform, CDDSs significantly enhance therapeutic efficacy while effectively mitigating risks associated with off-target effects.

Despite demonstrating substantial potential in treating ocular neovascular diseases, CDDSs face multiple challenges in clinical translation (Table 1 summarizes the advantages and limitations of the various CDDS platforms discussed in this review). First, the synergistic mechanisms among multiple modules remain poorly understood. The spatiotemporal coordination among targeting ligands, responsive elements, and drug release kinetics lacks systematic investigation. Potential interference among functional modules may constrain the maximization of therapeutic efficacy. Resolving this issue requires sophisticated material design and systematic in vitro–in vivo correlation studies. Second, reliable real-time monitoring methods are lacking. Current assessments of drug distribution and release kinetics predominantly rely on ex vivo tissue analysis or in vitro simulated release, failing to accurately reflect dynamic processes within the living ocular environment. This technical gap limits the in-depth understanding of the in vivo fate of carriers, necessitating the development of non-invasive real-time tracking technologies combining advanced imaging (e.g., optical coherence tomography and fluorescence imaging) with smart nanosensors. Additionally, long-term biosafety data accumulation remains insufficient. While short-term studies confirm the preliminary safety of existing systems, issues such as chronic toxicity after repeated dosing, carrier residue, metabolic pathways of degradation products, and potential immunogenicity require clarification through long-term animal model experiments and systematic biocompatibility evaluations. At the clinical translation level, sustained-release systems and minimally invasive delivery devices based on FDA-approved biodegradable materials (e.g., PLGA and lipids) have successfully entered clinical use, representing the mainstream pathway for CDDS clinical translation. Representative products include dexamethasone intravitreal implants (Ozurdex^®^) [141], fluocinolone intravitreal implants (e.g., Iluvien^®^ and Retisert^®^) [142,143], and Visudyne^®^ [61] liposomes. The success of these systems validates the clinical value of CDDSs in extending dosing intervals and improving patient compliance. In contrast, smart response and multifunctionally integrated systems remain predominantly in preclinical or early clinical exploration phases. While demonstrating excellent targeting and controlled drug release capabilities in animal models, their complex manufacturing processes, lack of quality control standards, difficulties in scaling production, and insufficient long-term safety data have constrained their clinical advancement.

Although CDDSs face multiple challenges in clinical translation for ocular neovascular diseases, their academic value and application potential as innovative delivery platforms are widely recognized. Through continuous interdisciplinary innovation and systematic solutions to current limitations, these smart drug delivery systems hold immense potential to transform the therapeutic landscape of ocular neovascular diseases. Ultimately, they may deliver more effective and patient-centered treatments for vision-threatening conditions.

## 5. Conclusions

This review systematically outlines research progress in CDDSs for treating ocular neovascular diseases. This article first analyzes the anatomical and physiological barriers of the eye and the unique pathological microenvironment of neovascular lesions. It focuses on CDDS design strategies such as sustained-release formulations, stimulus-responsive platforms (ROS/enzyme/pH-sensitive), active targeting, and multifunctionally integrated systems, discussing the advantages and limitations of each platform. From a clinical translation perspective, nanoparticle delivery systems based on FDA-approved materials (e.g., PLGA and lipids) represent the platforms closest to clinical application. Their advantages include well-established material safety, mature manufacturing processes, and support for minimally invasive administration. In contrast, smart response and multifunctionally integrated systems, while representing future directions, face challenges such as design complexity, lack of quality control standards, and unclear long-term safety profiles. With breakthroughs in materials science and improvements in safety evaluation systems, next-generation smart response CDDSs are expected to gradually achieve clinical translation, offering more precise and safer therapeutic strategies for ocular neovascular diseases.

## Figures and Tables

**Table 1:** Advantages and limitations of CDDSs for ocular drug delivery.

CDDS Type	Triggers	Advantages	Limitations
Controlled-Release CDDSs	Passive diffusion/degradation: Polymer hydrolysis Liposomal membrane permeability	Established safety profile: Utilize FDA-approved materials with proven biocompatibility. Prolonged therapeutic effect: Extend drug half-life and ocular residence time. Industrial scalability: Amenable to scale-up via simple, well-established manufacturing processes. Broad drug compatibility: Encapsulate both hydrophilic and lipophilic agents via aqueous core and lipid bilayer.	Burst release risk: Initial rapid release may compromise pharmacokinetic control. Invasive administration: Require intravitreal injection, hindering patient adherence. Limited targeting: Lack active pathological tissue recognition, relying on passive mechanisms. Formulation challenges: Liposomal systems suffer from instability, low encapsulation efficiency, short half-life, and sterilization hurdles.
ROS-Responsive CDDSs	Pathologically high ROS levels	Smart controlled release: Achieve site-specific drug release in pathological high-ROS microenvironments with high spatial selectivity. Therapeutic synergy: Carrier materials possess intrinsic ROS-scavenging capacity, enabling combined drug release and antioxidant effects. Versatile administration routes: Adaptable to intravitreal (MSNs), ocular surface (hydrogels), and periocular (microneedles) delivery.	Unreliable response kinetics: Complex in vivo milieu may compromise ROS-responsive efficacy and release kinetics. Manufacturing complexity: Multifunctional modifications complicate synthesis and challenge batch-to-batch reproducibility. Pathological variability: Heterogeneity in ROS levels across patients and disease stages restricts universal applicability. Unclear biosafety: Long-term fate and toxicity of inorganic materials in ocular tissues remain poorly characterized.
Enzyme-Responsive CDDSs	Specific enzymes overexpressed in pathological lesions (e.g., MMP-2/9 in neovascular tissues)	Enzyme-triggered specificity: Leverage disease-associated enzymes (e.g., MMP-2/9) for precise site-specific drug release. Multimodal synergy: Integrate targeting, responsive release, and ROS scavenging within a single platform (e.g., DNA origami) for amplified therapeutic efficacy. In situ morphological transition: GPNT systems convert from nanoparticles to nanofibers at target sites, enabling sustained drug retention.	Premature degradation risk: Enzyme-sensitive linkers susceptible to non-specific cleavage in complex ocular enzymatic environments. Biomacromolecule instability: Peptide/DNA components exhibit poor in vivo stability, requiring stabilization strategies. Immunogenicity potential: Exogenous macromolecular platforms (e.g., DNA origami) may provoke undesired immune responses, compromising safety/efficacy. Inherent manufacturing complexity: Multi-step surface functionalization complicates process control and hinders scalable production.
pH-Responsive CDDSs	Acidic microenvironments	Leveraging acidic microenvironments: Exploit pathological pH gradients for site-specific drug release. Enhanced corneal permeability: pH-sensitive materials undergo charge reversal or conformational changes to improve mucosal penetration. Programmable sequential release: Microneedle systems enable tailored multi-drug delivery (e.g., rapid release followed by sustained action).	Narrow response window: Limited pH fluctuation range on the ocular surface imposes stringent requirements on material sensitivity and precision of the pH-responsive threshold. Premature drug leakage: Inadequate stability under physiological pH (7.4) may result in significant drug loss before reaching target tissues. Restricted applicability: Primarily suitable for anterior segment diseases (e.g., infectious keratitis and glaucoma) with limited utility for posterior segment disorders.
Multifunctionally Integrated CDDSs	Multi-modal combination: Endogenous (pH/Enzyme/ROS) + exogenous (light/magnetic/heat) Can respond to multiple signals simultaneously	Synergistic efficacy: Combine targeting, controlled release, and microenvironment modulation. Overcoming multiple barriers: Designed to address penetration, retention, and cellular targeting in a single platform.	Formidable manufacturing complexity: Multi-step functionalization (e.g., membrane coating and peptide modification) hinders scale-up and GMP compliance. Protracted safety assessment: Multi-component nature confounds evaluation of long-term toxicity, immunogenicity, and biodegradation. Regulatory ambiguity: Complex architecture poses challenges for FDA/EMA approval pathways. Prohibitive development cost: Sophisticated materials and processing limit clinical accessibility and commercial viability.

## Data Availability

No new data were created or analyzed in this study. Data sharing is not applicable to this article.
